# OMRT-3. Longitudinal analysis of diffuse glioma reveals cell state dynamics at recurrence associated with changes in genetics and the microenvironment

**DOI:** 10.1093/noajnl/vdab070.029

**Published:** 2021-07-05

**Authors:** Frederick Varn, Kevin Johnson, Floris Barthel, Hoon Kim, Taylor Wade, Tathiane Malta, Thais Sabedot, Disha Lodha, Shoaib Ajaib, Nazia Ahmed, Luciano Garofano, Fulvio D’Angelo, Lucy Stead, Laila Poisson, Houtan Noushmehr, Antonio Iavarone, Roel Verhaak, GLASS Consortium

**Affiliations:** 1 The Jackson Laboratory for Genomic Medicine, Farmington, CT, USA; 2 University of Sao Paulo, Ribeirao Preto, Brazil; 3 Department of Neurosurgery, Henry Ford Health System, Henry Ford Cancer Institute, Detroit, MI, USA; 4 Leeds Institute of Medical Research at St James’s, University of Leeds, Leeds, UK; 5 Institute for Cancer Genetics, Columbia University Medical Center, New York, NY, USA; 6 Department of Biostatistics, Henry Ford Health System, Henry Ford Cancer Institute, Detroit, MI, USA; 7 Department of Neurology, Columbia University Medical Center, New York, NY, USA

## Abstract

Diffuse glioma is an aggressive brain cancer that is characterized by a poor prognosis and a universal resistance to therapy. The evolutionary processes behind this resistance remain unclear. Previous studies by the Glioma Longitudinal Analysis (GLASS) Consortium have indicated that therapy-induced selective pressures shape the genetic evolution of glioma in a stochastic manner. However, single-cell studies have revealed that malignant glioma cells are highly plastic and transition their cell state in response to diverse challenges, including changes in the microenvironment and the administration of standard-of-care therapy. Interactions between these factors remain poorly understood, making it difficult to predict how a patient’s tumor will evolve from diagnosis to recurrence. To interrogate the factors driving therapy resistance in diffuse glioma, we collected and analyzed RNA- and/or DNA-sequencing data from temporally separated tumor pairs of 292 adult patients with IDH-wild-type or IDH-mutant glioma. Recurrent tumors exhibited diverse changes that were attributable to changes in anatomic composition, somatic alterations, and microenvironment interactions. Hypermutation and acquired *CDKN2A* homozygous deletions associated with an increase in proliferating stem-like malignant cells at recurrence in both glioma subtypes, reflecting active tumor expansion. IDH-wild-type tumors were more invasive at recurrence, and their malignant cells exhibited increased expression of neuronal signaling programs that reflected a possible role for neuronal interactions in promoting glioma progression. Mesenchymal transition was associated with the presence of a specific myeloid cell state defined by unique ligand-receptor interactions with malignant cells, providing opportunities to target this transition through therapy. Collectively, our results uncover recurrence-associated changes in genetics and the microenvironment that can be targeted to shape disease progression following initial diagnosis.

